# An Inactivated Antibiotic-Exposed Whole-Cell Vaccine Enhances Bactericidal Activities Against Multidrug-Resistant *Acinetobacter baumannii*

**DOI:** 10.1038/srep22332

**Published:** 2016-02-29

**Authors:** Meng-Hooi Shu, NorAziyah MatRahim, NurAsyura NorAmdan, Sui-Ping Pang, Sharina H. Hashim, Wai-Hong Phoon, Sazaly AbuBakar

**Affiliations:** 1 Tropical Infectious Diseases Research and Education Centre, Department of Medical Microbiology, Faculty of Medicine, University of Malaya, 50603 Kuala Lumpur, Malaysia; 2 Virology Unit, Institute for Medical Research, 50588 Kuala Lumpur, Malaysia

## Abstract

Vaccination may be an alternative treatment for infection with multidrug-resistance (MDR) *Acinetobacter baumannii*. The study reported here evaluated the bactericidal antibody responses following immunization of mice using an inactivated whole-cell vaccine derived from antibiotic-exposed MDR *A. baumannii* (I-M28-47-114). Mice inoculated with I-M28-47 (non-antibiotic-exposed control) and I-M28-47-114 showed a high IgG antibody response by day 5 post-inoculation. Sera from mice inoculated with I-M28-47-114 collected on day 30 resulted in 80.7 ± 12.0% complement-mediated bacteriolysis *in vitro* of the test MDR *A. baumannii* treated with imipenem, which was a higher level of bacteriolysis over sera from mice inoculated with I-M28-47. Macrophage-like U937 cells eliminated 49.3 ± 11.6% of the test MDR *A. baumannii* treated with imipenem when opsonized with sera from mice inoculated with I-M28-47-114, which was a higher level of elimination than observed for test MDR *A. baumannii* opsonized with sera from mice inoculated with I-M28-47. These results suggest that vaccination with I-M28-47-114 stimulated antibody responses capable of mounting high bactericidal killing of MDR *A. baumannii*. Therefore, the inactivated antibiotic-exposed whole-cell vaccine (I-M28-47-114) has potential for development as a candidate vaccine for broad clearance and protection against MDR *A. baumannii* infections.

*Acinetobacter baumannii* is a strictly aerobic, non-fastidious and non-motile gram-negative coccobacillus. Over the past few decades, the bacteria has emerged as one of the major causes of healthcare facility-acquired nosocomial infections^1^,^2^. The bacterium is associated with bloodstream infection (septicaemia), surgical site infections, wound infections and brain and spinal cord infections (meningitis). *A. baumannii* can also be community-acquired, resulting mainly in respiratory tract infections (pneumonia) and wound infections, especially in unusual situations such as victims of natural disasters and wars^3^,^4^. Infections in critically ill patients, such as those requiring the use of ventilator, can be deadly. Factors influencing predisposition to *A. baumannii* infections include the use of invasive devices such as mechanical ventilation, previous long-term use of broad-spectrum antibiotics, major surgery, burns, wounds and immunosuppression.

Rapid acquisition of resistance to diverse classes of antibiotics has made treatment of *A. baumannii* infections difficult. Carbapenems have been the antibiotic of choice for the treatment of *A. baumannii* infections. However, resistance to this antibiotic has been increasingly reported and has reached up to 80% in many European healthcare facilities^5^,^6^,^7^. Due to the difficulty in treating multidrug-resistance (MDR) *A. baumannii* infections, novel approaches to prevention or treatment are needed. Vaccination may be an alternative approach to combating this pathogen^8^,^9^.

To date, there are no licensed vaccines against *A. baumannii*. Live-attenuated and inactivated whole-cell bacteria have been used for the development of a number of vaccine candidates due to their ability to induce protective immune responses^8^,^10^,^11^,^12^,^13^. It has been suggested that inactivated whole-cell vaccines could be safer than the live-attenuated vaccines^8^,^14^. Here, we developed an inactivated whole-cell vaccine derived from antibiotic-exposed MDR *A. baumannii*. The antibiotic pre-treatment of the MDR *A. baumannii* was previously shown to enhance the expression of *A. baumannii* proteins conferring resistance to the antibiotics. We investigated whether this newly developed vaccine approach enhances the efficacy and potential protective immunity against *A. baumannii*, especially the MDR strains, by evaluating the ability of the vaccine to improve the complement-mediated and macrophage-mediated bactericidal activities of the immunized sera.

## Results

### Antibody responses after inoculation

**Figure 1:**
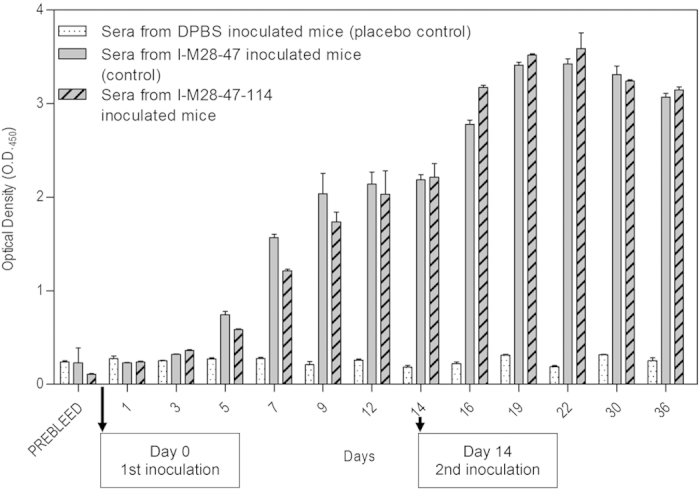
Kinetics of the IgG antibody response to inoculation with I-M28-47 and I-M28-47-114. The levels of antigen-specific IgG were determined by indirect ELISA in pooled sera from mice inoculated with I-M28-47-114, I-M28-47 (control) or DPBS (placebo control) and challenged on day 14. The data are expressed as the mean ± S.D. absorbance units.

Immunized mice sera were collected on days 0 to 36, and the humoral immune response was evaluated using an indirect enzyme-linked immunosorbent assay (ELISA). IgG antibody was detected in mice inoculated with I-M28-47 (inactivated whole-cell vaccine derived from non-antibiotic-exposed bacteria) and I-M28-47-114 (inactivated whole-cell vaccine derived from antibiotic-exposed bacteria) as early as on day 5 with optical density (O.D.) values of 0.74 ± 0.04 and 0.58 ± 0.02, respectively (Fig. 1). On day 14, the O.D. values of 2.18 ± 0.06 and 2.21 ± 0.25 were observed in mice inoculated with I-M28-47 and I-M28-47-114, respectively, showing an increase in IgG antibody. The O.D. values of 3.43 ± 0.05 and 3.59 ± 0.29 were detected in sera of mice collected after a second inoculation on day 22 with I-M28-47 and I-M28-47-114, respectively, which resulted in a rapid memory response peak. The O.D. values on day 36 in mice inoculated with I-M28-47 and I-M28-47-114 was observed to be 3.07 ± 0.04 and 3.15 ± 0.06, respectively, showing a slight decrease in the antibody response. The sera from placebo-inoculated control mice (Dulbecco’s phosphate buffered saline, DPBS) contained very low levels of detectable antibodies at any time-point with an average O.D. value of 0.30 ± 0.03.

### Complement-mediated bacteriolysis activity

**Figure 2:**
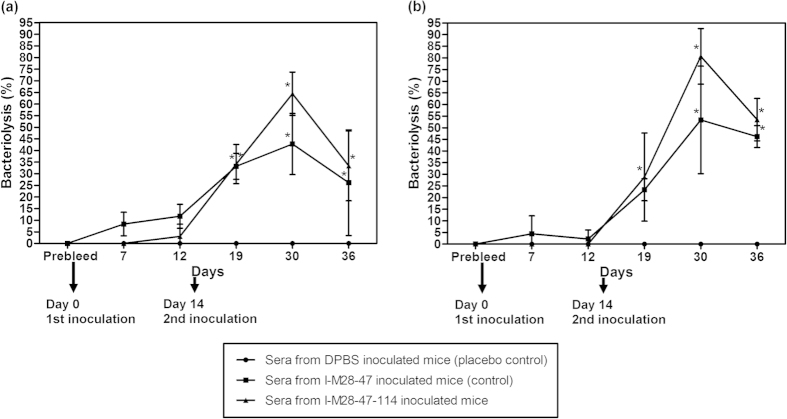
Complement-mediated bacteriolysis activity of sera from mice inoculated with I-M28-47 and I-M28-47-114 against two different MDR *A. baumannii* growth conditions. The lysis activity percentages were determined using sera from mice inoculated with I-M28-47-114, I-M28-47 (control) or DPBS (placebo control) in presence of baby rabbit complement against MDR *A. baumannii* (**a**) cultured without imipenem treatment or (**b**) treated with 32 mg/L imipenem. The values are the means ± S.D. tested in duplicate. *P < 0.05 represents significant differences in lysis between the vaccine groups and the placebo control group.

Immunized mice sera collected on days 0, 7, 12, 19, 30 and 36 were tested for the ability to promote *in vitro* complement-mediated killing activity of the test MDR *A. baumannii*. The average number of test MDR *A. baumannii* colonies cultured without imipenem treatment on agar plates after treatment with the placebo-treated control mice sera was 1.88 × 10^9^ ± 3.04 × 10^8^ cfu/ml (Fig. 2). The test MDR *A. baumannii* treated with 32 mg/L imipenem resulted 4.78 × 10^8^ ± 2.07 × 10^8^ cfu/ml of *A. baumannii* colonies when treated with the placebo-treated control mice sera.

Sera of mice from the first inoculation with I-M28-47 and I-M28-47-114 collected on days 7 and 12 resulted in the highest killing percentage of only 11.7 ± 5.2% of test MDR *A. baumannii* cultured without imipenem treatment (Fig. 2a). The percentage killing of test MDR *A. baumannii* treated with imipenem was between 0% to 4.4 ± 7.7% after treatment with the sera of mice inoculated with I-M28-47 and I-M28-47-114 collected on days 7 and 12 (Fig. 2b).

The sera of mice collected after the second inoculation on day 30 from the I-M28-47 inoculation group resulted in 42.8 ± 13.2% killing of the test MDR *A. baumannii* cultured without imipenem treatment, which was a significant (*P* < 0.01) increase in the bacteriolysis activity (Fig. 2a). The sera of mice collected on day 30 from the I-M28-47-114 inoculation group showed 64.4 ± 9.3% bacteriolysis activity against the test MDR *A. baumannii* cultured without imipenem treatment. When tested against the MDR *A. baumannii* treated with imipenem, the sera collected on day 30 from the I-M28-47 inoculation group resulted in 53.3 ± 23.1% killing (Fig. 2b). A killing percentage of 80.7 ± 12.0% was observed with sera collected on day 30 from the I-M28-47-114 inoculation group when used against the test MDR *A. baumannii* treated with imipenem, demonstrating a significant (*P* < 0.01) increase in the bacteriolysis activity.

The sera of mice collected on day 36 from I-M28-47 and I-M28-47-114 inoculation groups showed 26.2 ± 22.7% and 33.4 ± 15.0% bacteriolysis activity against the test MDR *A. baumannii* cultured without imipenem treatment, respectively (Fig. 2a). Meanwhile, the percentage of bacteriolysis activity for the sera of mice inoculated with I-M28-47 and I-M28-47-114 collected on day 36 were at 46.2 ± 4.7% and 53.5 ± 9.1%, respectively, against the test MDR *A. baumannii* treated with imipenem (Fig. 2b).

### Opsonophagocytic killing activity of macrophage-like U937 or RAW 264.7 cells

Opsonophagocytic killing assays using the test MDR *A. baumannii* was used to assess whether the inoculation with I-M28-47 and I-M28-47-114 induces immune protection mediated by phagocytosis. The macrophage-like U937 and RAW 264.7 cell lines were used for these assays.

**Figure 3:**
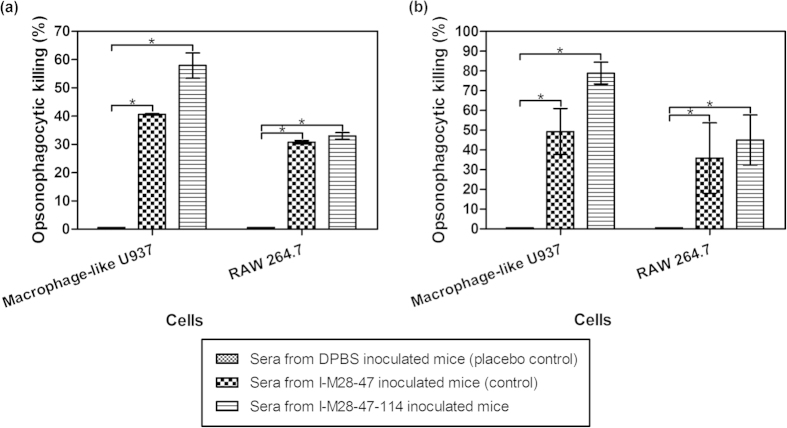
Percentage of opsonophagocytic killing activity following opsonisation with sera from mice inoculated with I-M28-47 and I-M28-47-114. Opsonization enhances the uptake and killing of MDR *A. baumannii* (**a**) cultured without imipenem treatment or (**b**) treated with 32 mg/L imipenem by macrophage-like U937 and RAW 264.7 cells. All the values are the means ± S.D. tested in duplicate. A significant difference in killing between the vaccine groups and the placebo control groups was observed (*P < 0.05).

For the macrophage-like U937 cells, the opsonophagocytic killing activity of test MDR *A. baumannii* without imipenem treatment or treated with 32 mg/L imipenem and opsonized with sera collected on day 36 from placebo-inoculated control mice showed averages of 1.18 × 10^9^ ± 1.41 × 10^6^ cfu/ml and 7.91 × 10^8^ ± 1.56 × 10^7^ cfu/ml of *A. baumannii* colonies, respectively (Fig. 3). Specific opsonins present in the immunized mice sera collected on day 36 were detectable following I-M28-47 and I-M28-47-114 inoculation, wherein, showing a phagocytic killing of 40.6 ± 0.2% and 57.9 ± 4.5% of the test MDR *A. baumannii* without imipenem treatment, respectively (Fig. 3a). This resulted in a significant (*P* < 0.001) reduction of approximately 2.5 times of the number of test *A. baumannii* colonies (6.98 × 10^8^ ± 2.83 × 10^6^ cfu/ml and 4.95 × 10^8^ ± 5.23 × 10^7^ cfu/ml) in comparison to the placebo-inoculated control mice (1.18 × 10^9^ ± 1.41 × 10^6^ cfu/ml). Test MDR *A. baumannii* treated with imipenem-specific opsonins present in the sera of mice collected on day 36 after I-M28-47 inoculation resulted in a phagocytic killing of 49.3 ± 11.6%, in which almost half of the test MDR *A. baumannii* growth was significantly (*P* < 0.001) eliminated (Fig. 3b). The percentage of phagocytic killing of test MDR *A. baumannii* treated with imipenem using sera of mice from the I-M28-47-114 inoculation group collected on day 36 was at 78.9 ± 5.5%, which was a significantly (*P* < 0.001) higher increase in comparison to the sera of mice from the I-M28-47 inoculation group.

The opsonophagocytic killing assay was repeated using RAW 264.7 cells. The average number of *A. baumannii* colonies was 1.22 × 10^9^ ± 2.23 × 10^8^ cfu/ml and 9.83 × 10^8^ ± 4.10 × 10^7^ cfu/ml when the test MDR *A. baumannii* without imipenem treatment or treatment with 32 mg/L imipenem, respectively, were opsonized with sera collected on day 36 from placebo-inoculated control mice (Fig. 3). The sera collected on day 36 from mice inoculated with I-M28-47 and I-M28-47-114 showed an almost parallel percentage of opsonophagocytic killing of 30.8 ± 0.6% and 33.0 ± 1.2% against the test MDR *A. baumannii* without imipenem treatment, respectively (Fig. 3a). In contrast, the sera collected on day 36 from mice inoculated with I-M28-47 showed 35.9 ± 17.8% phagocytic killing against test MDR *A. baumannii* treated with imipenem (Fig. 3b). The sera collected on day 36 from mice inoculated with I-M28-47-114 resulted in phagocytic killing of 45.1 ± 12.7% against the test *A. baumannii* treated with imipenem, which was a significantly higher level (*P* < 0.01) of phagocytic killing in comparison to sera from mice inoculated with I-M28-47.

### Cross-reactivity of immunized mice sera

The possible cross-reactivity of the immunized mice sera against another gram-negative bacterium in terms of the ability to promote *in vitro* complement-mediated killing and opsonophagocytic killing activity was examined using *Escherichia coli* (*E. coli*).

For complement-mediated bacteriolysis activity, the immunized mice sera collected on days 30 and 36 from the I-M28-47 and I-M28-47-114 inoculation group were used and compared against the sera from the placebo-inoculated control mice. The sera collected on day 30 from the I-M28-47 and I-M28-47-114 inoculation groups, when used against *E. coli*, resulted in only 2.7 ± 3.8% and 3.3 ± 2.8% killing, respectively, which was a non-significant increase in the bacteriolysis activity (Table 1). The sera of mice collected on day 36 from the I-M28-47 and I-M28-47-114 inoculation groups showed only 4.1 ± 3.1% and 1.4 ± 2.3% bacteriolysis activity against *E. coli*, respectively.

For opsonophagocytic killing activity of RAW 264.7 cells against *E. coli*, the immunized mice sera collected on day 36 from the I-M28-47 and I-M28-47-114 inoculation group were used and compared against sera from the placebo-inoculated control mice. The sera collected on day 36 from the mice inoculated with I-M28-47 and I-M28-47-114, resulted in 0% and 4.8 ± 6.8% phagocytic killing, respectively, against *E. coli* (Table 1).

## Discussion

Over the past few decades, *A. baumannii* has emerged as a major healthcare facility-acquired bacterial infection. The infection is particularly problematic among individuals on long-term mechanical ventilation, on previous broad-spectrum antibiotic therapy, with previous stays in intensive care units or long-term care facilities, having previously undergone major surgery, or among patients with burns, wounds, immunosuppression and military personal with battle-acquired wounds^3^,^4^. Treatment of *A. baumannii* infections has also become a clinical challenge due to the ever-increasing resistance of the bacteria to diverse classes of antibiotics. Targeted vaccination represents a possible alternative to circumvent the serious infections caused by this pathogen. A recent study demonstrated that an inactivated whole-cell vaccine against *A. baumannii* was effective in preventing dissemination of *A. baumannii* infection^8^. Here, we used an inactivated whole-cell vaccine derived from antibiotic-exposed MDR *A. baumannii* (I-M28-47-114) to determine if this newly developed candidate vaccine can enhance the efficacy and protective immunity against *A. baumannii*, especially the MDR strains. We tested the I-M28-47-114 vaccine in comparison to the inactivated non-antibiotic-exposed whole-cell vaccine (I-M28-47), which was derived from the same MDR *A. baumannii* strain. The antibody responses elicited by the newly developed vaccine (I-M28-47-114) was evaluated, and the ability of the vaccine to improve killing of MDR *A. baumannii* by complement-mediated bacteriolysis and/or opsonophagocytic killing may reflect potential protection against infection.

The findings from this study suggest that a robust IgG antibody response to *A. baumannii* was present in the sera from mice inoculated with the I-M28-47-114 and I-M28-47, whereas the sera from the placebo-inoculated control mice (DPBS) expressed very low detectable IgG antibodies. Inoculation with I-M28-47 and I-M28-47-114 stimulated high levels of serum IgG antibody recognizing the MDR *A. baumannii* strain used as the target antigen. These results are consistent with an earlier study where inactivated whole-cell vaccine derived from *A. baumannii* was used as a vaccine^8^. Together, these findings, support the potential development of a vaccine against MDR *A. baumannii* using inactivated whole-cell bacteria.

Although there are extensive data on the immune responses following vaccination with inactivated whole-cell vaccines derived from *A. baumannii*^8^,^9^,^15^, the possible mechanisms of clearance or protection against *A. baumannii* infections mediated by the antibodies have not been well described. In the present study, we showed that the sera obtained from mice inoculated with I-M28-47 and I-M28-47-114 were able to activate complement-mediated bacteriolysis of MDR *A. baumannii*. It was noted that the bacteriolysis effects in sera of mice from the first inoculation with I-M28-47 and I-M28-47-114 collected at days 7 and 12 showed only a slight incremental increase, suggesting that sera from a single immunization did not significantly increase the complement-mediated bacteriolysis activity. The results after the booster immunization suggested that the two vaccine groups were able to mount effective responses, resulting in inactivation of the MDR *A. baumannii*. The bacteriolysis effects were sustained up to day 30 post-inoculation with the vaccines. The complement-mediated bacteriolysis effect in the sera collected on day 30 from mice inoculated with I-M28-47-114 was much higher in comparison to the sera collected on the same day from mice inoculated with I-M28-47 against MDR *A. baumannii* treated with imipenem. The lysis effect in the sera collected on day 30 from mice inoculated with I-M28-47-114 was also found to be higher in the MDR *A. baumannii* treated with imipenem in comparison to the test *A. baumannii* cultured without imipenem treatment. These results suggest that inoculation with I-M28-47-114 stimulated an immune response that mediated significantly better clearance or bacteriolysis activity of MDR *A. baumannii* in comparison to I-M28-47 inoculation. No such effect was observed in the sera from placebo-inoculated control mice, although the low IgG level was noted within this group. In the presence of low-level or absent of specific antibodies, not surprisingly, the mice sera did not kill *A. baumannii*. This is consistent with other studies that have shown that *A. baumannii* cannot activate complement *in vitro* without the presence of a specific antibody response^16^,^17^,^18^. Activation of the complement pathway is an important immune defence mechanism present in serum. Hence, complement activation could play an important role in minimizing the ability of *A. baumannii* to escape immune clearance.

In our study, we demonstrated that opsonization with antibodies from mice inoculated with I-M28-47 and I-M28-47-114 augmented both the uptake and killing of *A. baumannii* by macrophage-like U937 and RAW 264.7 cells. The two macrophage cell lines were used as different macrophage cells may act differently in terms of phagocytosis. Our findings, using the placebo control group, suggest that the macrophages could not mediate phagocytosis of *A. baumannii* in the presence of low-level or absent of specific antibody against the bacteria. In comparison, opsonisation with sera from the two vaccine groups, I-M28-47 and I-M28-47-114, showed significant macrophage-mediated bacterial killing. The results also suggest that the antibodies from mice inoculated with I-M28-47-114 significantly mediated higher uptake and killing by macrophage-like U937 and RAW 264.7 cells in comparison to the sera from mice inoculated with I-M28-47 against MDR *A. baumannii* treated with imipenem. The recruitment of macrophages to the site of initial infection is an important immune defence against a bacterial infection. The coating and binding of specific antibodies onto the surface of bacteria is a prerequisite for macrophages to promote efficient recognition for bacterial ingestion.

Our findings here are consistent with earlier studies, which suggest that a specific antibody response stimulated by an inactivated whole-cell vaccine can aid in the clearance of *A. baumannii* and accord protection against *A. baumannii* infections^8^,^16^,^17^,^19^. The inactivated whole-cell vaccine has been suggested as a viable approach as this type of vaccine has the advantage of presenting multiple antigens of the bacteria to the immune system. This aspect could be crucial against a possible broad range of bacterial strains within the *A. baumannii* species. The findings from our study represent a significant step forward in delineating the functional roles of antibodies in the protection against MDR *A. baumannii*. However, the antibody type or subtype responsible for the clearance of *A. baumannii* or protection against *A. baumannii* infections has not been characterized due to limited serum availability. Further work is still necessary to address the type or subtype of antibody and the possible antigens that are responsible for the bacterial killing effects. In addition, we currently do not know which MDR *A. baumannii* proteins are antigenic and inducible in the presence of the antibiotic imipenem, was used to prepare the vaccine in this study.

The findings described in our study demonstrated that antibodies from mice immunized with the I-M28-47-114 inactivated whole-cell vaccine derived from imipenem-exposed MDR *A. baumannii* elicited efficient killing of MDR *A. baumannii* through the complement-mediated bacteriolysis and phagocytosis pathways. We demonstrated that a vaccine against MDR *A. baumannii* using an antibiotic-exposed bacterial culture stimulated higher immunogenicity than a vaccine not exposed to an antibiotic. In addition, we demonstrated that the vaccine developed in the study is specific against MDR *A. baumannii* and is not cross-reactive against *E. coli*, another gram-negative bacterium. This vaccine development approach may potentially further the development of viable vaccines against MDR *A. baumannii* infections.

## Materials and Methods

### Bacterial strains

The *A. baumannii* clinical isolate strain M28-47 was previously isolated^20^ at the University Malaya Medical Centre. The isolate was stored at −80 °C as stock cultures and was subcultured twice onto Mueller-Hinton (MH) agar plates (BD Biosciences, New Jersey, United States) prior to use as previously described^20^. The full bacterial genome was sequenced and stored for future reference.

### Antimicrobial susceptibility test

A loop of *A. baumannii* was inoculated into DPBS (Sigma-Aldrich, Missouri, United States) until an 0.5 McFarland standard (Biomérieux, Marcy-l'Étoile, France) turbidity was obtained. The suspension was then swabbed onto MH agar plates. Next, 11 impregnated antibiotic discs (BD Biosciences, New Jersey, United States) or an Epilometer test (E-test) strip containing a gradient of impregnated imipenem concentrations (Biomérieux, Marcy-l'Étoile, France) were placed on the surface of the agar, and the plates were incubated at 37 °C overnight. The disc diffusion zone of inhibition was measured and interpreted based on the Clinical and Laboratory Standards Institute^21^. The isolate was resistant to all of the 11 antibiotics (Supplementary Table 1). The E-test strip was read at the intersection of the zone edge and the strip, and the values were interpreted as minimal inhibitory concentrations (MICs). The E-test MIC of imipenem was 32 mg/L (Supplementary Table 1).

### Preparation of the inactivated antibiotic-exposed whole-cell bacteria vaccine (I-M28-47-IMP)

A loop of *A. baumannii* was inoculated into MH broth medium (BD Biosciences, New Jersey, United States) and was cultured overnight at 37 °C with continuous gentle shaking. The following day, the bacterial culture was transferred (1:24) into MH broth medium and incubated at 37 °C with continuous gentle shaking until it reached early log phase at an O.D._600_ = 0.3. Freshly prepared imipenem (Merck Sharp and Dohme, New Jersey, United States) at the MIC concentration (32 mg/L) was added into the bacterial culture. The culture was continuously incubated until it reached late log phase at O.D._600_ = 0.8. Medium containing the bacterial culture (1:24) without the imipenem treatment was prepared in parallel to serve as the non-antibiotic-exposed control. Following the incubation, the bacterial cultures were centrifuged at 3,000 × *g* for 15 minutes. The supernatants were discarded, and the cell pellets were washed twice with DPBS. Subsequently, the bacterial cells were inactivated with DPBS containing 3% formaldehyde (Merck, Darmstadt, Germany) for 2 hours at room temperature and for an additional 16 hours at 60 °C with constant gentle shaking. Before inactivation, the bacterial concentrations were determined by plating the supernatant onto MH agar plates and incubation overnight at 37 °C. Following the inactivation step, the bacterial suspensions were sedimented at 3,000 × *g* for 15 minutes. The supernatant was discarded, and the bacterial pellets were rigorously washed twice with DPBS. DPBS was then added to the cell pellet (corresponding to 10^6^ cfu/ml) and was stored frozen at −80 °C. Complete inactivation of the bacteria was confirmed by plating the supernatant onto MH agar. The inactivated whole-cell bacteria preparations, I-M28-47-114 (antibiotic-exposed) and I-M28-47 (non-antibiotic-exposed), were used as the vaccines in this study. The I-M28-47-114 and I-M28-47 vaccines were preserved in 2.5% 2-phenoxyethanol diluted in DPBS at a 4:1 ratio.

### Mice and inoculation schedule

Adult male BALB/cJ mice between 6 to 8 weeks of age were purchased from the University of Malaya and housed under specific pathogen-free conditions. The animal experiments were performed in accordance with the protocols and guidelines approved by the University of Malaya Institutional Animal Care and Use Committee, Malaysia (Ethic Number: MP/10/04/2007/SAB(R)). The mice were randomly divided into 3 groups. Group 1 was vaccinated with I-M28-47-114, group 2 with DPBS (placebo), and group 3 with I-M28-47.

The vaccines and DPBS were mixed (1:1) with Freund’s adjuvant (Sigma-Aldrich, Missouri, United States). Complete Freund’s adjuvant was used for the first inoculation and incomplete Freund’s adjuvant was used for the second inoculation. The vaccine mixtures were inoculated into mice by intramuscular injection on days 0 and 14. The initial inoculation was administered after the pre-bleed serum (referred to as day 0 or pre-inoculation) was collected. The mice were then bled almost every other day (days 1, 3, 5, 7, 9, 12, 14, 16, 19, 22, 30 and 36) and sacrificed at the end of the experiment. The control group mice were similarly inoculated and bled.

### Assessment of antibody responses

The inactivated antibiotic-exposed whole-cell bacteria, I-M28-47-114 (corresponding to 10^7^ cfu/ml), was used as the antigen in the ELISA. The antigen was diluted 2:1 with carbonate buffer and was coated to the microtiter plates overnight at 4 °C. Following the coating step, the plates were washed with phosphate buffered saline (PBS) containing 0.05% Tween 20 (PBS-T) and were blocked with PBS-T containing 10% bovine serum albumin for 2 hours at room temperature. After the blocking step, the plates were washed three times with PBS-T. The diluted pooled immunized mice sera (1:50 with PBS) was then added and incubated for 2 hours at 37 °C. After washing, diluted alkaline phosphatase-conjugated goat anti-mouse antibodies specific for IgG (Sigma-Aldrich, Missouri, United States) (1:7,500 with PBS) was added and incubated for 2 hours at 37 °C. Following washing, the *p*-nitrophenylphosphate substrate was prepared following the manufacturer’s recommended protocol (KPL, Maryland, USA) was added to the plates. After 10 minutes, the O.D. reading was measured using a microplate reader (Tecan, Männedorf, Switzerland) at a wavelength of 450/520 nm.

### Complement-mediated bacteriolysis assay

Freshly prepared *A. baumannii* was inoculated into MH broth medium and was incubated overnight at 37 °C with continuous gentle shaking. The following day, the bacterial culture was transferred (1:100) into MH broth medium and was incubated at 37 °C with continuous gentle shaking. At early log phase (O.D._600_ = 0.3) imipenem at 32 mg/L was added to the bacterial culture. The culture was incubated until it reached late log phase (O.D._600_ = 0.8). At the end of the incubation, the bacterial culture was serially diluted to 1:1,000,000 (corresponding to 100–200 live bacteria) in DPBS. An aliquot of the diluted bacterial culture was added to 5% heat-inactivated pooled immunized mice sera (30 minutes at 56 °C) and 5% baby rabbit complement (Pel-Freez Biologicals, Arkansas, USA). The mixture was incubated for 1 hour at 37 °C, plated on MH agar, incubated overnight at 37 °C, and the number of bacterial colonies was determined. A bacterial culture maintained in parallel but without the imipenem treatment was used for comparison purposes. The percentage of bacteriolysis was calculated as:


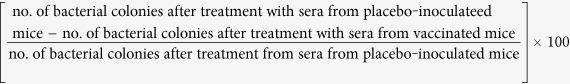


### Opsonophagocytic killing assay

Human U937 promonocytic cells (American Type Culture Collection (ATCC), Virginia, USA) were used in these studies and were maintained as previously described^22^. The cells were seeded in Roswell Park Memorial Institute-1640 medium (RPMI-1640) (Gibco, Massachusett, USA) with 2% heat-inactivated FBS, and differentiation was induced (to macrophage-like cells) the following day with 10 nM phorbol 12-myristate 13-acetate (PMA) (Sigma-Aldrich, Missouri, USA) for 48 hours as previously described^22^. The mouse leukaemic monocyte macrophage cell line, RAW 264.7 cells (ATCC, Virginia, USA), were maintained in Dulbecco’s modified Eagle medium (DMEM) (Gibco, Massachusett, USA) containing 1% 10 mM non-essential amino acid (NEAA) (Hyclone, Utah, USA) and 1% 10 mM L-glutamine (L-Glu) (Hyclone, Utah, USA) with 10% heat-inactivated foetal bovine serum (FBS) (Hyclone, Utah, USA) at 37 °C in a humidified incubator with 5% CO_2_.

Macrophage-like U937 or RAW 264.7 cells were seeded into 96-well cell culture plates (Falcon, Fisher Scientific, Pennsylvania, USA) at a density of 2 × 10^4^ cells/well in RPMI-1640 or DMEM with 2% FBS, respectively. The cells were incubated overnight at 37 °C in a humidified incubator with 5% CO_2_. The following day, the cell culture medium containing non-adherent cells was removed before performing the phagocytosis assay.

For the opsonization assay, a loop of *A. baumannii* culture was inoculated into MH broth medium and was incubated overnight at 37 °C with continuous gentle shaking. The following day, the bacterial culture was transferred into MH broth medium and was incubated as described above. Imipenem at 32 mg/L was added to the bacterial culture at early log phase (O.D._600_ = 0.3) and was continuously incubated until reaching late log phase (O.D._600_ = 0.8). At the end of the incubation, the bacterial culture was serially diluted with DPBS to 1:100,000. A bacterial culture maintained in parallel but without imipenem treatment was used for comparison purposes. Heat-inactivated pooled immunized mice sera (5%) was added to aliquots of the diluted bacterial culture and were incubated for 30 minutes at 37 °C. These opsonized bacteria were used in the subsequent phagocytosis assay.

For the phagocytosis assay, the opsonized *A. baumannii* bacteria were overlaid on the macrophage cells prepared above. The cells were incubated for 1 hour at 37 °C in a humidified incubator with 5% CO_2_. Every 15 minutes, the plates were gently agitated for 5 minutes at room temperature. Non-phagocytosed *A. baumannii* were removed, plated on MH agar plates, were incubated overnight at 37 °C, and the number of bacterial colonies were determined. Percentage of killing was calculated as:


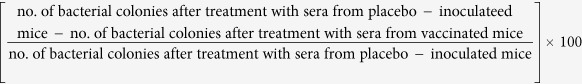


### Cross-reactivity of immunized mice sera against *E. coli*

A loop of *E. coli* strain NovaBlue T1^R^ (Novagen, Merck, Darmstadt, Germany) was inoculated into lysogeny broth (LB) medium (Oxoid, Thermo Fisher Scientific, Massachusetts, United States) and was cultured overnight at 37 °C with continuous gentle shaking. The following day, the bacterial culture was transferred (1:100) into LB broth medium and was incubated at 37 °C with continuous gentle shaking until it reached late log phase (O.D._600_ = 0.8). At the end of the incubation, the bacterial culture was serially diluted to 1:100,000 in DPBS.

For the complement-mediated bacteriolysis assay, an aliquot of the diluted bacterial culture was added to 5% heat-inactivated pooled immunized mice sera and 5% baby rabbit complement. The mixture was incubated as described above, plated on LB agar (Oxoid, Thermo Fisher Scientific, Massachusetts, United States), incubated overnight at 37 °C, and the number of bacterial colonies was determined. The percentage of bacteriolysis was calculated as described above.

For the opsonophagocytic killing assay, heat-inactivated pooled immunized mice sera (5%) was added to aliquots of the diluted bacterial culture and were incubated as described above. The opsonized *E. coli* was then overlaid on the RAW 264.7 macrophage cells as prepared above. The cells were incubated as described above, and every 15 minutes the plates were gently agitated for 5 minutes at room temperature. Non-phagocytosed *E. coli* were removed, plated on LB agar plates, incubated overnight at 37 °C, and the number of bacterial colonies were determined. The percentage of killing was calculated as described above.

### Statistical analysis

The data from the antibody response, complement-mediated bacteriolysis and opsonophagocytic killing assays were expressed as the mean ± standard deviation (S.D.) from repeated assays. The data were subjected to two-way analysis of variance (ANOVA) and Bonferroni’s post-test. Statistical significance was considered as *P* < 0.05. All the statistical analyses were performed using GraphPad Prism 4.0 Software (California, United States).

## Additional Information

**How to cite this article**: Shu, M.-H. *et al*. An Inactivated Antibiotic-Exposed Whole-Cell Vaccine Enhances Bactericidal Activities Against Multidrug-Resistant *Acinetobacter baumannii. Sci. Rep.* **6**, 22332; doi: 10.1038/srep22332 (2016).

## Supplementary Material

Supplementary Information

## Figures and Tables

**Table 1:** Complement-mediated bacteriolysis and opsonophagocytic killing activity of immunized mice sera against *E. coli*.

Sera from mice inoculated with:	Percentage of bacteriolysis (%)
Day 30	
DPBS (placebo control)	0.0 ± 0.0
I-M28-47 (control)	2.7 ± 3.8
I-M28-47-114	3.3 ± 2.8
Day 36	
DPBS	0.0 ± 0.0
I-M28-47	4.1 ± 3.1
I-M28-47-114	1.4 ± 2.3
**Sera from mice inoculated with:**	**Percentage of opsonophagocytic killing (%)**
Day 36	
DPBS (placebo control)	0.0 ± 0.0
I-M28-47 (control)	0.0 ± 0.0
I-M28-47-114	4.8 ± 6.8

The values are the means ± S.D. tested in duplicate. *P < 0.05 represents significant difference between the vaccine groups and the placebo control group.
