# Supplementary material for: An Inactivated Antibiotic-Exposed Whole-Cell Vaccine Enhances Bactericidal Activities Against Multidrug-Resistant Acinetobacter baumannii
Source: Sci Rep. 2016 Feb 29;6:22332. doi: 10.1038/srep22332 (PMC4770312; doi:10.1038/srep22332)
Supplement: Supplementary Information [file srep22332-s1.doc]

**An Inactivated Antibiotic-Exposed Whole-Cell Vaccine Enhances Bactericidal Activities against Multidrug-Resistant *Acinetobacter baumannii***

**Meng-Hooi Shu^1^, NorAziyah MatRahim^2^, NurAsyura NorAmdan^1^, Sui-Ping Pang^1^, Sharina H. Hashim^1^, Wai-Hong Phoon^1^, and Sazaly AbuBakar^1,*^**

^1^Tropical Infectious Diseases Research and Education Centre, Department of Medical Microbiology, Faculty of Medicine, University of Malaya, 50603 Kuala Lumpur, Malaysia

^2^Virology Unit, Institute for Medical Research, 50588 Kuala Lumpur

**Supplementary Table 1. Antimicrobial susceptibility of *A. baumannii*** strain M28-47.

| Antimicrobial agent | Disc diffusion zone of inhibition (mm) | E-test MICs (mg/L) |
| --- | --- | --- |
| 10 µg Ampicillin | R | - |
| 10 µg Gentamicin | R | - |
| 30 µg Amikacin | I | - |
| 30 µg Cefuroxime | R | - |
| 75 µg Cefoperazone | R | - |
| 30 µg Ceftazidime | R | - |
| 30 µg Ceftriaxone | R | - |
| 5 µg Ciprofloxacin | R | - |
| 30 µg Cefotaxime | R | - |
| 30 µg Amoxicillin-clavulanate | R | - |
| 10 µg Imipenem | R | 32 |

R, Resistance; S, Sensitive; I, Intermediate.
